# Supplementary figures and images for: Generation of cryopreserved macrophages from normal and genetically engineered human pluripotent stem cells for disease modelling
Source: PLoS One. 2021 Apr 22;16(4):e0250107. doi: 10.1371/journal.pone.0250107 (PMC8061979; doi:10.1371/journal.pone.0250107)

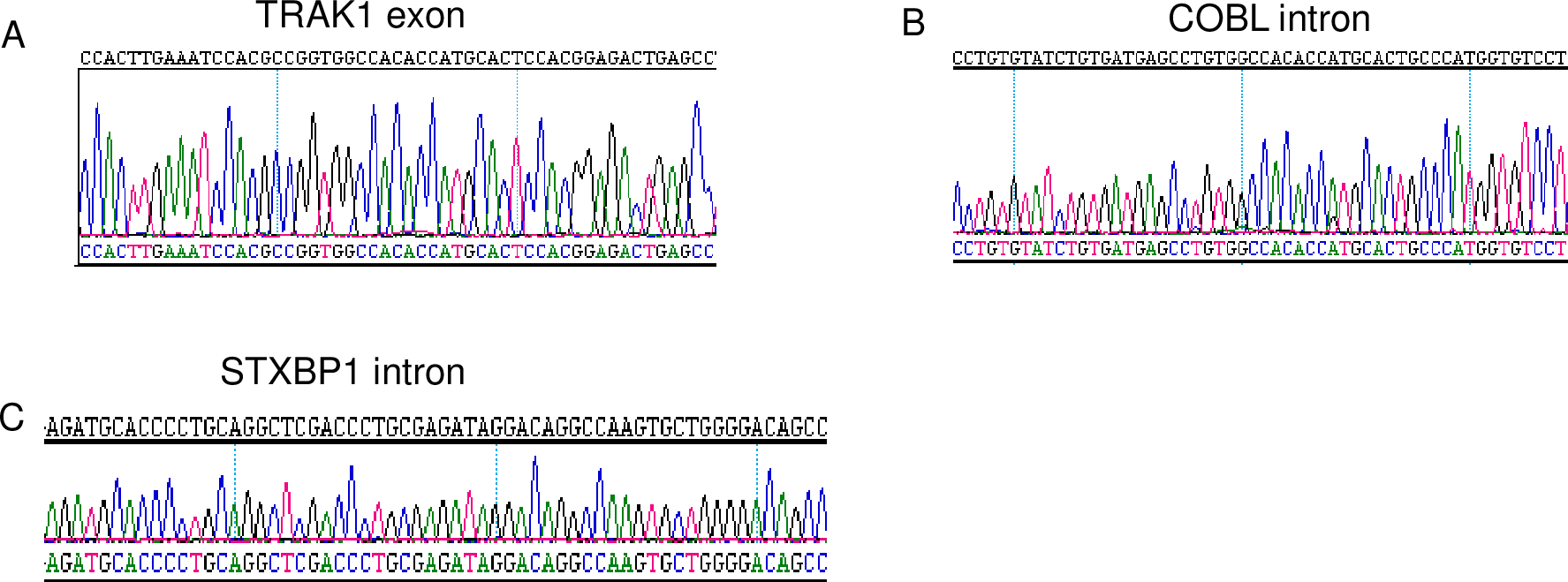

Supplement: S1 Fig — Amplification and sequencing was performed across the TRAK1 (A) or COBL (B) genes covering the two predicted “off-by-one” cut sites for the SNCA A53T nuclease. Sequence confirmation of no off-target mutations in GRN R493X HO iPSC. Amplification and sequencing was performed across the STXBP1 (C) gene at the predicted “off-by-one” cut site for the GRN R493X nuclease. (TIF) [file pone.0250107.s001.tif]

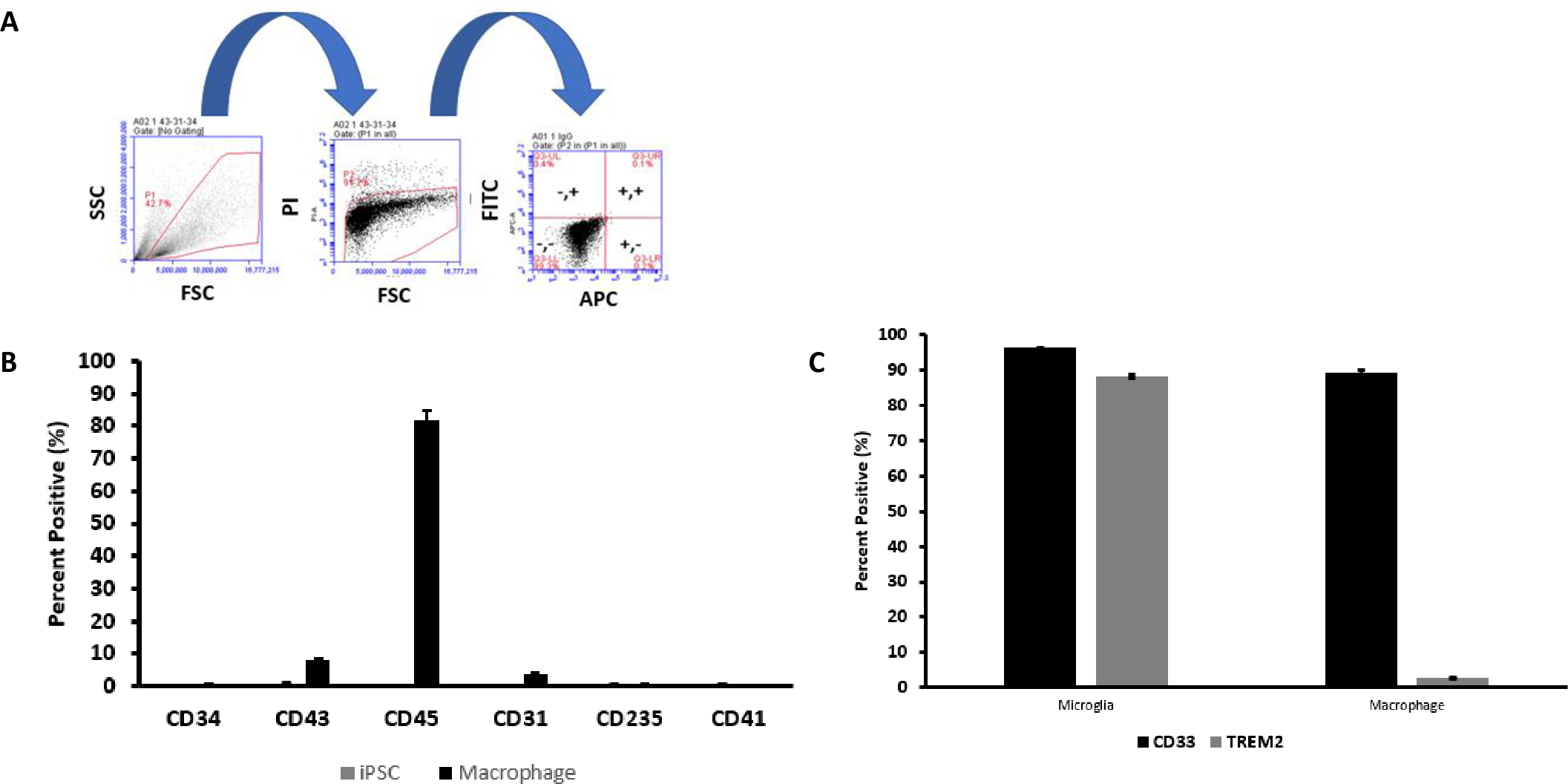

Supplement: S2 Fig — (A) Representative image of the gating strategy used during flowcytometric analysis. The sample is first scatter plat reflects the sample analysis using the FSC vs SSC gates. The live population is a subset of the population gated from the FSC vs. SSC gate stained with Propidium Iodine. Subsequent staining is gated on the live cells to determine single and double positive staining. (B) Parental iPSC and macrophages were stained for the presence of HPC associated markers. The graph denotes the absence of HPC markers on macrophages except CD45. Each value on the graph is an average of ± SE for iPSC and macrophages. (C) Comparative analysis of TREM2 and CD33 expression on iPSC derived microglia and iPSC derived macrophages. Graphs depicts average ± SE for microglia (n = 16) and macrophage (n = 3). (TIF) [file pone.0250107.s002.tif]

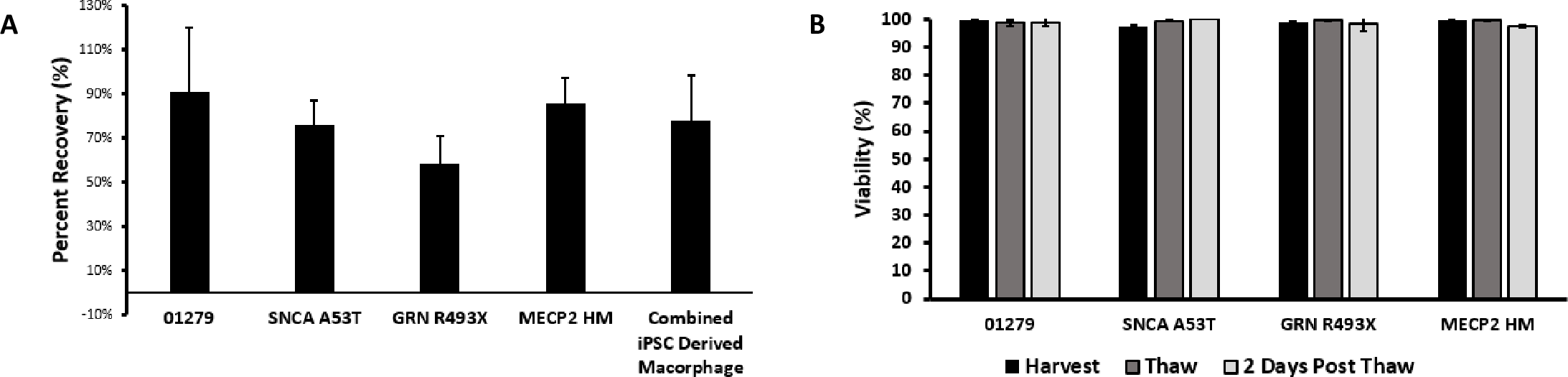

Supplement: S3 Fig — (A) Recovery was quantified by total viable cell number at cryopreservation divided by total viable cell number at thaw. The graphs denote recovery of iPSC derived macrophages post thaw for parental and isogenic disease lines (n = 4 ±SE) and average recovery of parental and engineered macrophages (n = 16 ±SE). (B) Viability of macrophages at harvest (n = 4 ±SE), upon thaw (n = 4 ±SE), and 2 days post thaw (n = 4 ±SE) was quantified by Trypan Blue exclusion on an automated cell counting system. (TIF) [file pone.0250107.s003.tif]

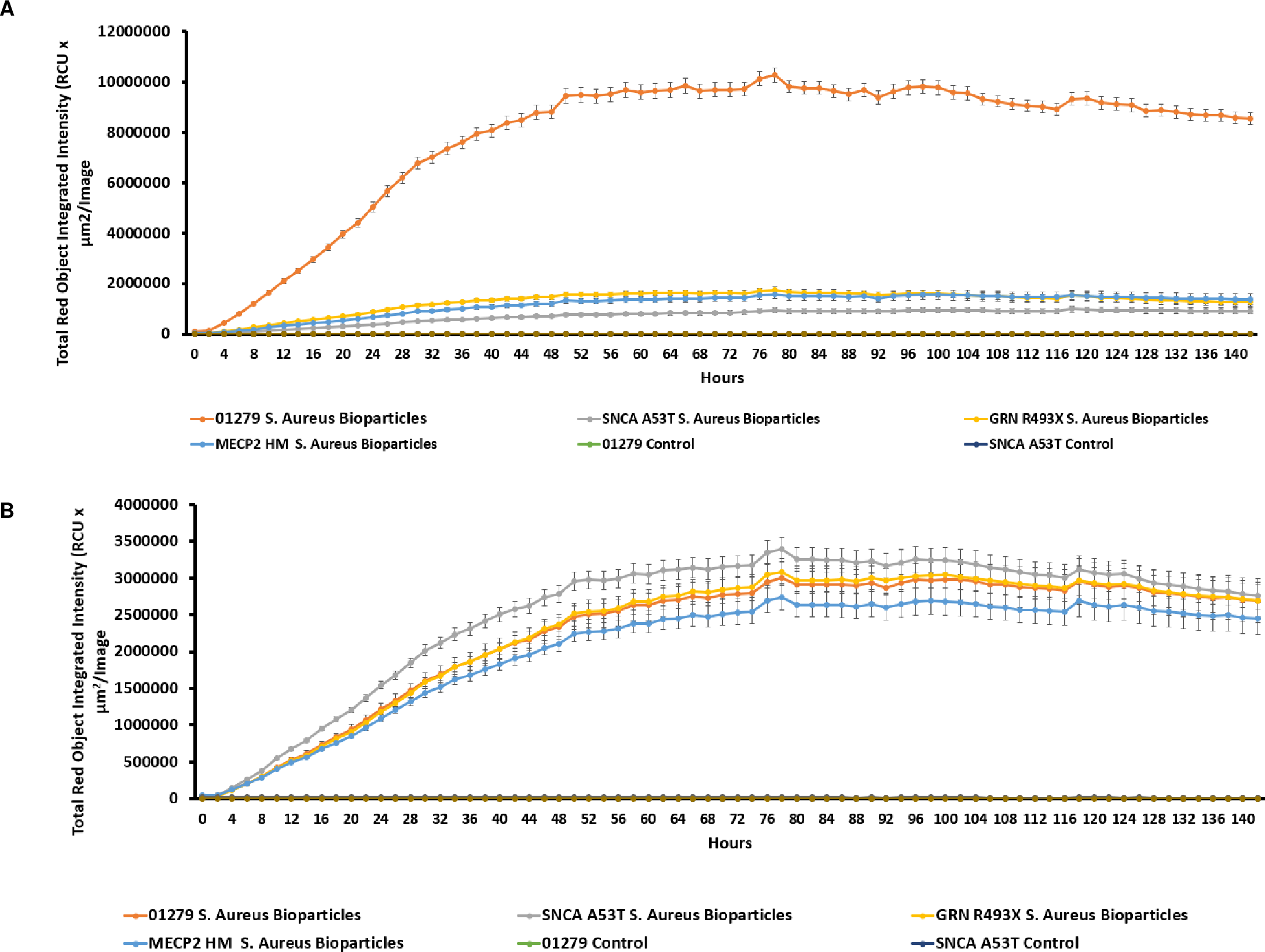

Supplement: S4 Fig — Phagocytotic function of 01279, SNCA A53T, GRN R493X, and MECP2 HM iPSC derived macrophages was measured by the red fluorescence intensity (RCU) captured on the IncuCyte S3 every 2 hours over the course of 6 days. Total Red Object Integrated Intensity (RCU x μm2/image) was analysed using IncuCyte Software (v2019B). The graphs depict the cumulative uptake of pHrodo labeled S. aureus bioparticles by live (n = 3 ± SE) (A) and cryopreserved (n = 19 ± SE) (B) macrophages. (TIF) [file pone.0250107.s004.tif]

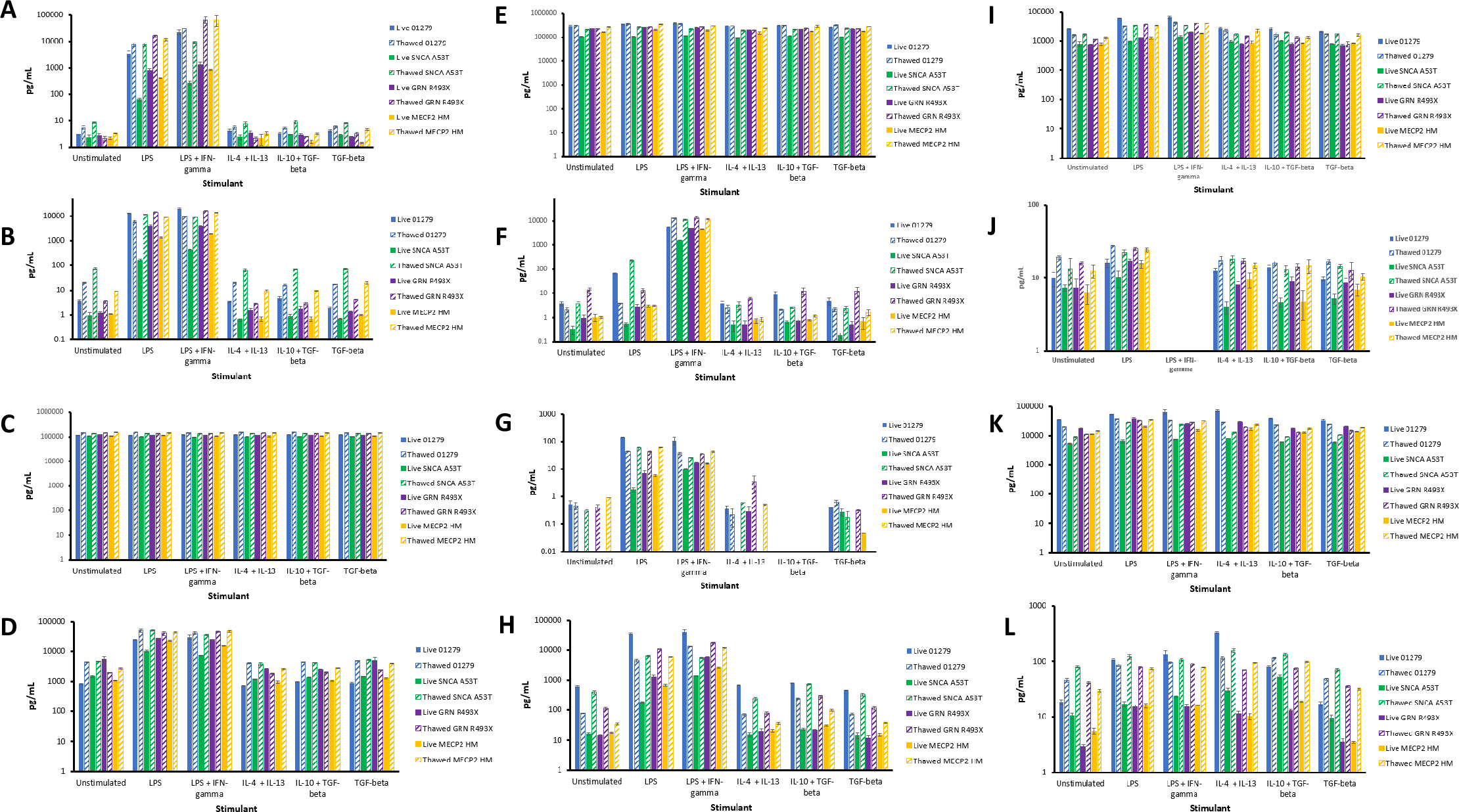

Supplement: S5 Fig — Live and cryopreserved macrophages (three days post thaw) were placed in cytokine free media and stimulated with LPS, LPS + Interferon Gamma, IL-4 + IL-13, IL-10 + TGF-Beta, and TGF-Beta for 24 hours. Release of analytes were quantified from the supernatants using Luminex multiplex system. Each graph depicts an average of triplicate samples ± SE for the specific analyte. The list of the specific analyte are as follows, TNF-alpha (A), IL-6 (B), MMP-9 (C), IL-8/CXCL8 (D), Chitinase 3-like 1 (E), CXCL10 (F), IL-10 (G), CCL2 (H), CD163 (I), IFN-gamma (J), IL-1ra (K), and CCL18 (L). (TIF) [file pone.0250107.s005.tif]

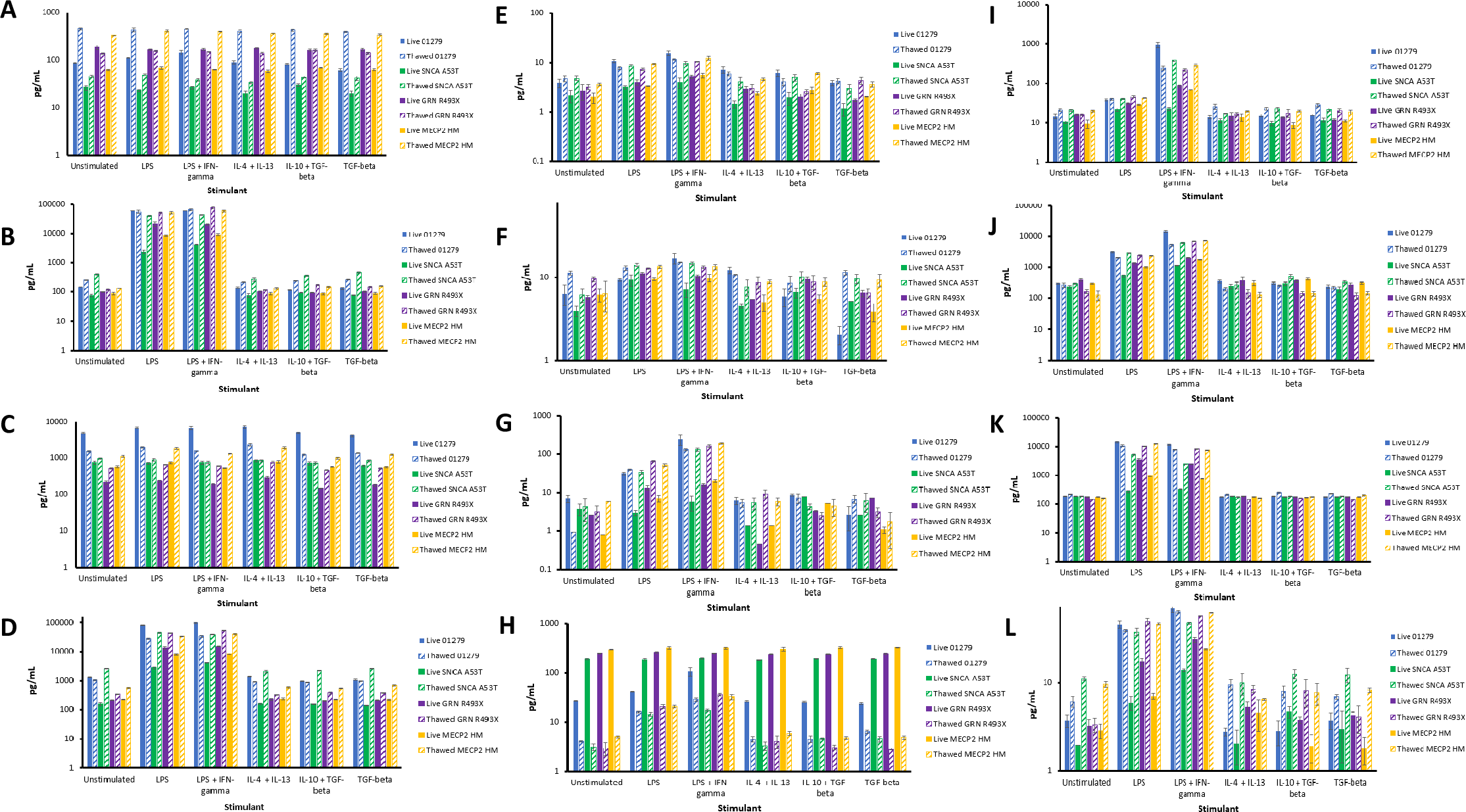

Supplement: S6 Fig — Live and cryopreserved macrophages (three days post thaw) were placed in cytokine free media and stimulated with LPS, LPS + Interferon Gamma, IL-4 + IL-13, IL-10 + TGF-Beta, and TGF-Beta for 24 hours. Release of analytes were quantified from the supernatants by Luminex multiplex system. Each graph depicts an average of triplicate samples ± SE for the specific analyte. The list of the specific analyte are as follows, MMP-12 (A), CCL3 (B), CCL22 (C), CCL4(D), IL-1 alpha(E), CCL26 (F), IL-12 p70 (G), IL-1 beta (H), CXCL11 (I), IL-23 (J), CXCL1 (K), and IL-18 (L). Each graph depicts an average of triplicate samples ± SE. (TIF) [file pone.0250107.s006.tif]

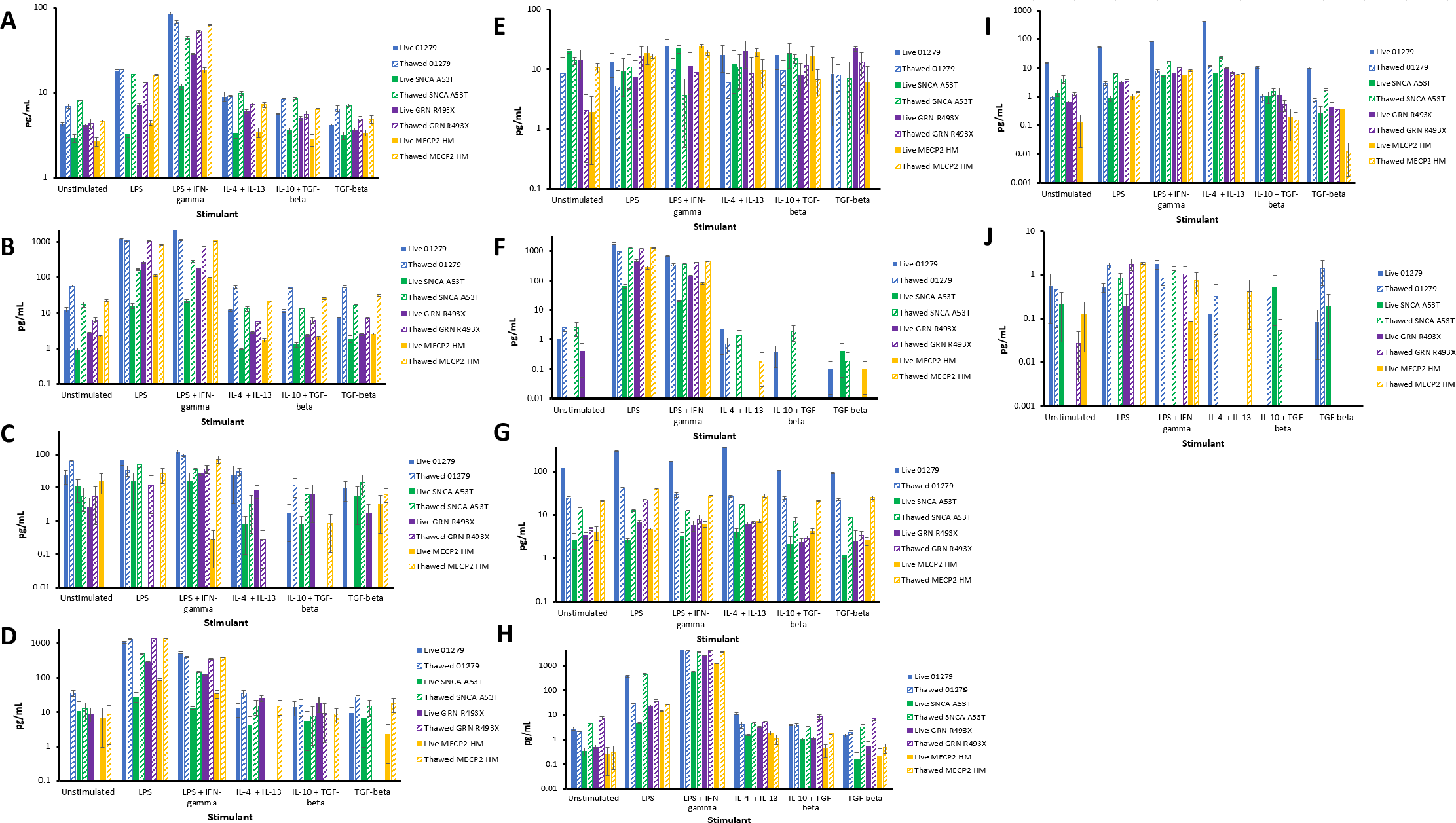

Supplement: S7 Fig — Live and cryopreserved macrophages (three days post thaw) were placed in cytokine free media and stimulated with LPS, LPS + Interferon Gamma, IL-4 + IL-13, IL-10 + TGF-Beta, and TGF-Beta for 24 hours. Release of analytes were quantified from the supernatants by Luminex multiplex system. Each graph depicts an average of triplicate samples ± SE for the specific analyte. The list of the specific analyte are as follows, PD-L1 (A), CCL1 (B), IL-27 (C), CXCL2 (D), CCL14 (E), CCL20 (F), CCL24 (G), CCL8 (H), CCL13 (I), and Fas Ligand (J). Each graph depicts an average of triplicate samples ± SE. (TIF) [file pone.0250107.s007.tif]

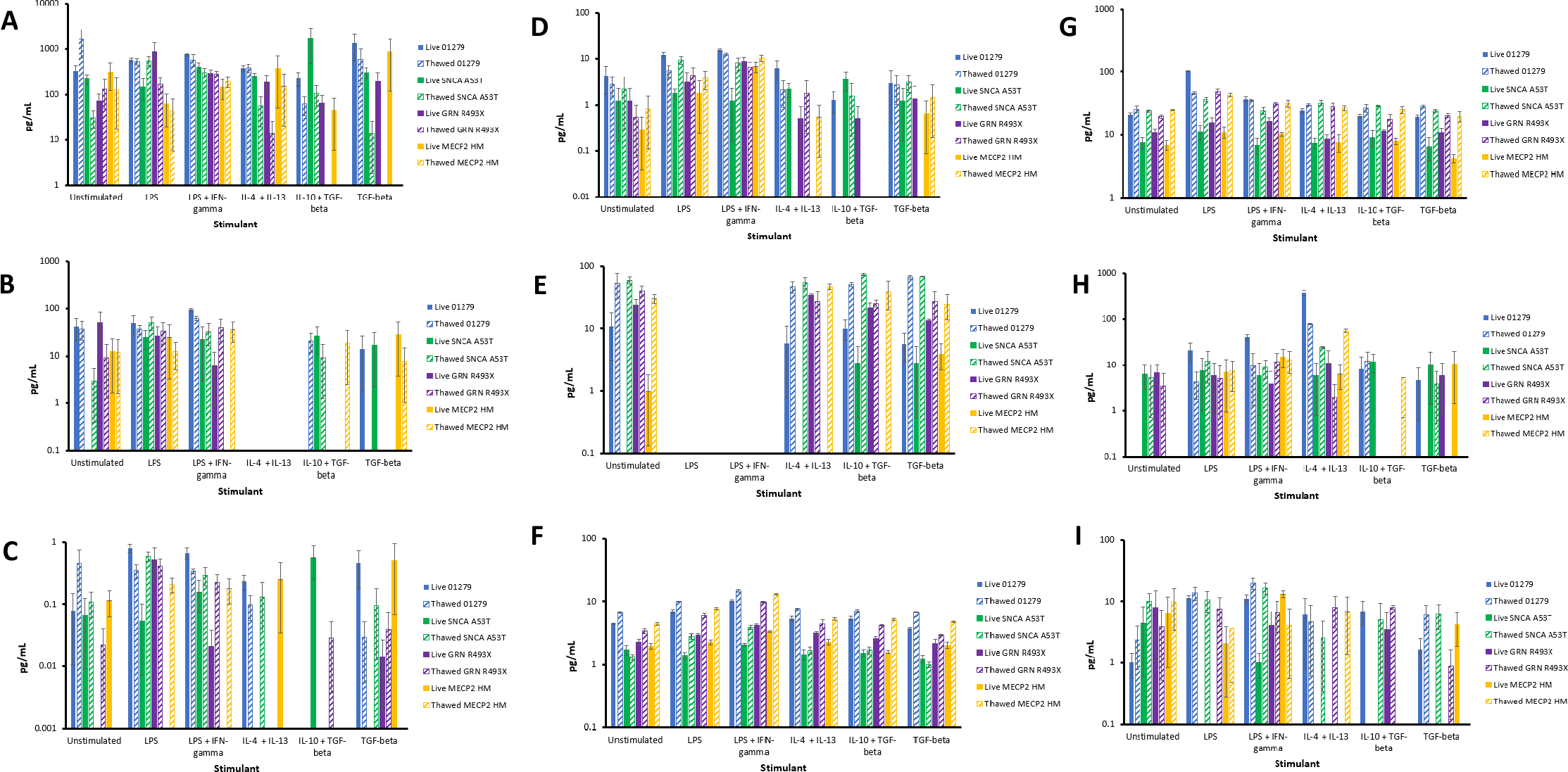

Supplement: S8 Fig — Live and cryopreserved macrophages (three days post thaw) were placed in cytokine free media and stimulated with LPS, LPS + Interferon Gamma, IL-4 + IL-13, IL-10 + TGF-Beta, and TGF-Beta for 24 hours. Release of analytes were quantified from the supernatants by Luminex multiplex system. Each graph depicts an average of triplicate samples ± SE for the specific analyte. The list of the specific analyte are as follows, CX3CL1/Fractalkine (A), IL-13 (B), TSLP (C), Granzyme B (D), Alpha-Synuclein (E), TGF-alpha (F), TREM1 (G), CCL17 (H), and CCL11 (I). Each graph depicts an average of triplicate samples ± SE. (TIF) [file pone.0250107.s008.tif]

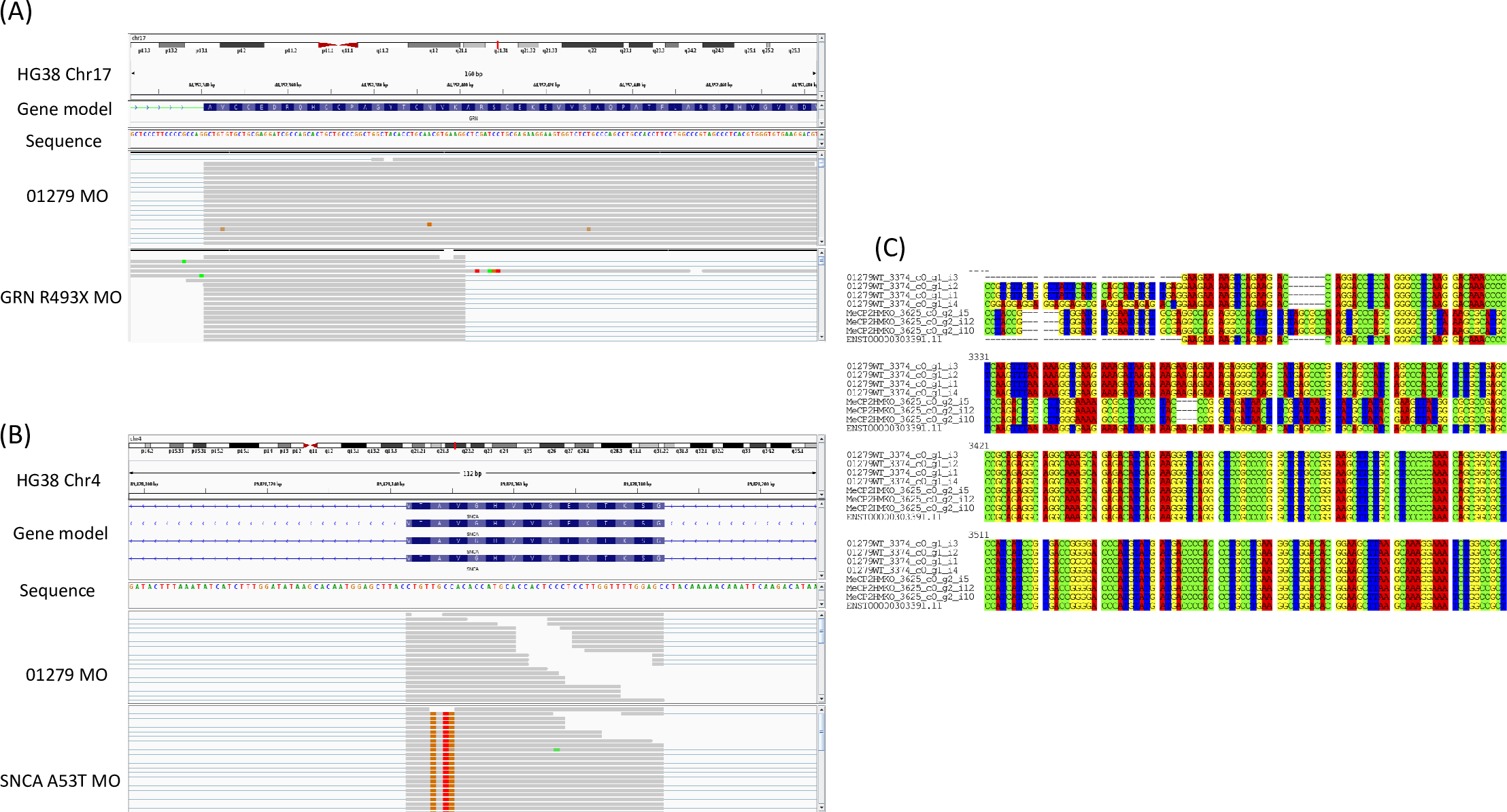

Supplement: S9 Fig — (A) Representative transcription tracks of 01279 (top panel) and GRN R493X (bottom panel) showing the truncated GRN transcription at amino acid 492. (B) Representative transcription tracks of 01279 (top panel) and SNCA A53T (bottom panel) showing the substitution at amino acid 53. (C) Multiple sequence alignment of assembled transcripts of 01279 and MeCP2 HM showing disrupted mutant transcripts after the puromycin insertion within exon3. (TIF) [file pone.0250107.s009.tif]
