# Supplementary material for: Generation of cryopreserved macrophages from normal and genetically engineered human pluripotent stem cells for disease modelling
Source: PLoS One. 2021 Apr 22;16(4):e0250107. doi: 10.1371/journal.pone.0250107 (PMC8061979; doi:10.1371/journal.pone.0250107)
Supplement: S1 Table — (DOCX) [file pone.0250107.s010.docx]

S1 Table: Oligonucleotides Used for Gene Editing

| **Primer** | **Sequence (5'-3')** | **Region and purpose** |
| --- | --- | --- |
| GRN_14-178 | CAGGTCTGGTTATCATGGCAG | GRN genotyping |
| GRN_14-179 | CGGCTGTGACCAGCACAC | GRN genotyping |
| SNCA_14-144 | caggaaacgtggagtacttac | SNCA genotyping |
| SNCA_14-145 | gacctcctgttagctgggct | SNCA genotyping |
| MeCP2_171 | TGAAGTGCGACTCATGCTGGG | MeCP2 screening |
| MeCP2_283 | attatgatccggctgcctcgcg | SV40pA screening |
| MeCP2_173 | TACGGTGCTCAGTCTCTCCAG | MeCP2 screening |
| MeCP2_218 | gccagaggccacttgtgtag | PGKp screening |
| TRAK1_15-5 | GTGTCCTGGGTCGTCCCTAC | TRAK1 genotyping |
| TRAK1_15-6 | CAGGGATGTGCTCATGGTGG | TRAK1 genotyping |
| COBL_15-11 | ctatgcacatgcagacacgacag | COBL genotyping |
| COBL_15-12 | tcaggtcagacaagagtggac | COBL genotyping |
| STXBP1_15-13 | GTAACTGCAACTCAGGCTGC | STXBP1 genotyping |
| STXBP1_15-14 | TACACATACACTGTGCTGGACTAC | STXBP1 genotyping |
| SJD 14-133 | GAAAACTAGCTAATCAGCAATTTAAGGCTAGCTTGAGACTTATGTCTTGAATTTGTTTTTGTAGGCTCCAAAACCAAGGAGGGAGTGGTGCATGGTGTCACCACAGGTAAGCTCCATTGTGCTTATATCCAAAGATGATATTTAAAGTATCTAGTGATTAGTGTGGCCCAGTATTCAAGATTCCTATGAAATTGTAAAAC | SNCA A53T donor oligo |
| SJD 14-180 | ACATAATGCCATTCTGTGCTCCCTTCCCCGCCAGGCTGTGTGCTGCGAGGATCGCCAGCACTGCTGCCCGGCTGGCTACACCTGCAACGTGAAGGCTTGAAGTTGCGAGAAGGAAGTGGTCTCTGCCCAGCCTGCCACCTTCCTGGCCCGTAGCCCTCACGTGGGTGTGAAGGACGTGGAGTGTGGGGAAGGACACTTCT | GRN R493X donor oligo |
