# Supplementary material for: Generation of cryopreserved macrophages from normal and genetically engineered human pluripotent stem cells for disease modelling
Source: PLoS One. 2021 Apr 22;16(4):e0250107. doi: 10.1371/journal.pone.0250107 (PMC8061979; doi:10.1371/journal.pone.0250107)
Supplement: S2 Table — (DOCX) [file pone.0250107.s011.docx]

S2 Table: Analyses of Surface and Intracellular Pluripotency Expression of Parental and Genetically Engineered iPSC Lines.

|  | **01279 (n=4)** | | **SNCA A53T (n=4)** | | **GRN R493X (n=4)** | | **MECP2 HM (n=4)** | |
| --- | --- | --- | --- | --- | --- | --- | --- | --- |
|  | **Mean** | **SE** | **Mean** | **SE** | **Mean** | **SE** | **Mean** | **SE** |
| **Tra160** | 90.8 | 2.9 | 84.2 | 7.3 | 92.6 | 1.1 | 92 | 2.1 |
| **CD90** | 99.7 | 0.5 | 99.7 | 0.6 | 100 | 0 | 91.5 | 0.5 |
| **SSEA4** | 99.4 | 0.8 | 98.1 | 0.2 | 98.8 | 0.5 | 93.4 | 0.5 |
| **SOX2** | 99.4 | 0.3 | 99.5 | 0.4 | 99.2 | 0.5 | 99.8 | 0.1 |
| **OCT3/4** | 89.4 | 5.4 | 90.4 | 5.5 | 91.9 | 3.7 | 93.5 | 2.1 |
| **NANOG** | 91 | 3.5 | 92.6 | 3.4 | 92 | 3 | 95.7 | 1.4 |
