# Supplementary material for: Generation of cryopreserved macrophages from normal and genetically engineered human pluripotent stem cells for disease modelling
Source: PLoS One. 2021 Apr 22;16(4):e0250107. doi: 10.1371/journal.pone.0250107 (PMC8061979; doi:10.1371/journal.pone.0250107)
Supplement: S3 Table — (DOCX) [file pone.0250107.s012.docx]

S3 Table: Mean and Standard Error of Colony Forming Unit (CFU) Assay for Parental and Genetically Engineered iPSC Lines.

|  | **01279 (n=32)** | | **SNCA A53T (n=6)** | | **GRN R493X (n=6)** | | **MECP2 HM (n=6)** | |
| --- | --- | --- | --- | --- | --- | --- | --- | --- |
|  | **Mean** | **SE** | **Mean** | **SE** | **Mean** | **SE** | **Mean** | **SE** |
| **CFU-E** | 3531.3 | 949.9 | 3566.7 | 363 | 3933.3 | 294.4 | 4100 | 707.1 |
| **BFU-E** | 7787.5 | 1919.6 | 6800 | 579.2 | 8333.3 | 496.7 | 3700 | 1272.8 |
| **CFU-M** | 3600 | 951.7 | 3666.7 | 536.6 | 3133.3 | 454.6 | 4500 | 707.1 |
| **CFU-G** | 1412.5 | 786.9 | 1300 | 255.2 | 2000 | 141.4 | 4600 | 848.5 |
| **CFU-GM** | 1187.5 | 340.5 | 833.3 | 178.7 | 1800 | 282.8 | 3200 | 565.7 |
| **GEMM** | 325 | 110.7 | 200 | 81.6 | 200 | 141.4 | 300 | 141.4 |
