# Supplementary material for: Generation of cryopreserved macrophages from normal and genetically engineered human pluripotent stem cells for disease modelling
Source: PLoS One. 2021 Apr 22;16(4):e0250107. doi: 10.1371/journal.pone.0250107 (PMC8061979; doi:10.1371/journal.pone.0250107)
Supplement: S4 Table — (DOCX) [file pone.0250107.s013.docx]

S4 Table: Mean and Standard Error of Macrophage Characterization of Cell Surface Antigen Expression.

|  | **01279 (n=3)** | | **SNCA A53T (n=3)** | | **GRN R493X (n=3)** | | **MECP2 HM KO (n=3)** | |
| --- | --- | --- | --- | --- | --- | --- | --- | --- |
|  | **Mean** | **SE** | **Mean** | **SE** | **Mean** | **SE** | **Mean** | **SE** |
| **CD64** | 97.13 | 0.50 | 95.83 | 0.32 | 99.30 | 0.10 | 97.50 | 0.20 |
| **CD33** | 89.40 | 0.78 | 96.37 | 0.23 | 95.20 | 0.30 | 88.30 | 1.35 |
| **CD11c** | 99.83 | 0.06 | 99.57 | 0.06 | 99.80 | 0.00 | 99.87 | 0.06 |
| **CD11b** | 98.63 | 0.06 | 97.80 | 0.53 | 99.20 | 0.26 | 99.13 | 0.06 |
| **CD80** | 84.00 | 0.82 | 87.17 | 1.23 | 87.73 | 1.79 | 84.27 | 1.36 |
| **CD169** | 48.37 | 2.29 | 74.00 | 1.01 | 64.83 | 4.41 | 59.23 | 6.01 |
| **CD206** | 82.20 | 1.65 | 90.27 | 0.25 | 90.70 | 1.73 | 81.30 | 2.81 |
| **CD86** | 79.13 | 0.72 | 91.57 | 0.95 | 89.33 | 0.80 | 85.50 | 0.36 |
| **CD1a** | 62.30 | 2.95 | 68.83 | 0.47 | 70.43 | 3.90 | 70.43 | 2.17 |
| **CD45** | 98.60 | 1.84 | 99.63 | 0.15 | 97.63 | 1.57 | 98.07 | 3.00 |
| **HLA-DR** | 22.00 | 30.55 | 30.55 | 11.47 | 26.90 | 15.48 | 27.73 | 18.24 |
| **CD340** | 27.83 | 4.35 | 44.17 | 2.75 | 33.40 | 7.65 | 49.57 | 1.80 |
| **CD163** | 16.67 | 1.15 | 19.57 | 1.63 | 16.30 | 1.75 | 19.37 | 1.75 |
| **CD14** | 0.00 | 0.00 | 0.00 | 0.00 | 0.00 | 0.00 | 0.00 | 0.00 |
| **CD47** | 99.90 | 0.00 | 99.90 | 0.00 | 99.90 | 0.00 | 99.90 | 0.00 |
| **HLA-ABC** | 83.30 | 0.44 | 86.93 | 0.50 | 90.00 | 1.30 | 82.83 | 0.42 |
| **CX3CR** | 73.63 | 0.67 | 70.83 | 4.38 | 93.73 | 0.50 | 77.80 | 0.53 |
| **TREM2** | 2.63 | 0.29 | 2.13 | 0.29 | 5.10 | 0.46 | 1.80 | 0.10 |
| **CD38** | 4.23 | 1.20 | 4.30 | 0.26 | 1.73 | 0.12 | 2.47 | 0.12 |
| **CD68** | 89.70 | 6.37 | 96.67 | 0.23 | 95.83 | 0.55 | 85.20 | 4.53 |
