# Supplementary material for: Generation of cryopreserved macrophages from normal and genetically engineered human pluripotent stem cells for disease modelling
Source: PLoS One. 2021 Apr 22;16(4):e0250107. doi: 10.1371/journal.pone.0250107 (PMC8061979; doi:10.1371/journal.pone.0250107)
Supplement: S5 Table — (a) Statistically significant overlapping differentially expressed genes within SNCA A53T iPSC derived macrophage. (b) Statistically significant overlapping differentially expressed genes within GRN R493X iPSC derived macrophage. (c) Statistically significant overlapping differentially expressed genes within MECP2 HM iPSC derived macrophage. (DOCX) [file pone.0250107.s014.docx]

S5a Table: Statistically Significant Overlapping Differentially Expressed Genes Within SNCA A53T iPSC derived macrophage

S5b Table: Statistically Significant Overlapping Differentially Expressed Genes Within GRN R493X iPSC derived macrophage

S5c Table: Statistically Significant Overlapping Differentially Expressed Genes Within MECP2 HM iPSC derived macrophage
