# Supplementary material for: Generation of cryopreserved macrophages from normal and genetically engineered human pluripotent stem cells for disease modelling
Source: PLoS One. 2021 Apr 22;16(4):e0250107. doi: 10.1371/journal.pone.0250107 (PMC8061979; doi:10.1371/journal.pone.0250107)
Supplement: Attachment — Submitted filename: Response To Reviewers.docx [file pone.0250107.s024.docx]

Enclosed is the summary of all questions addressed by the reviewers and our response to each query:

Reviewer 1:

1. **For full data transparency, please deposit RNAseq data files in a public database (i.e. Geo database) and include the link in the material and method section as well as in the Data availability statement.**

Response: The GEO submission is currently in progress and the link will be shared once generated.

Reviewer 2:

1. **It appears that the authors may have missed characterizing expression of key transcripts in the study, namely GRN, MECP2 and SNCA, as commented in the first round of review.**

Response: Transcript tracks for each mutation was included within the supplemental section and address within the Material and Methods and Results Section of the manuscript.

2. **The discussion in response to Q31 is appreciated. I would suggest incorporating it in the revised manuscript text.**

Response: Text correlating to the response to Q31, from the first revision, has been incorporated into the Introduction section of the manuscript.

3. **There does not appear to be any discussion with respect to the gender of the male 01279 cells. It is important that the audience recognize that male iPSCs have been used. A sentence or two of description would be appreciated (especially in the context of Rett syndrome).**

Response: A brief description of the impact of the gender parental line in the context of Rett Syndrome has been included in the Material and Methods section.

4. **The color depictions in Fig. 7 are not defined as absolute fold change, or Log fold-change. Is “0” white? If so, it would be advisable to indicate that no change is indicated in white.**

Response: The results are displayed in fold change. White would indicate no change (0), and the colors red and blue reflect a higher (red) and lower (blue) levels of cytokines released. A more detailed description has been added to the legend for figure 7.

5. **RNAseq data should be deposited into a public database (GEO); information regarding the data should be included in Materials/Methods**.

Response: Please see response to Reviewer 1, question 1.
